# Role of protein phosphorylation in cell signaling, disease, and the intervention therapy

**DOI:** 10.1002/mco2.175

**Published:** 2022-11-03

**Authors:** Kun Pang, Wei Wang, Jia‐Xin Qin, Zhen‐Duo Shi, Lin Hao, Yu‐Yang Ma, Hao Xu, Zhuo‐Xun Wu, Deng Pan, Zhe‐Sheng Chen, Cong‐Hui Han

**Affiliations:** ^1^ Department of Urology, Xuzhou Central Hospital, Xuzhou Clinical School of Xuzhou Medical College The Affiliated Xuzhou Hospital of Medical College of Southeast University The Affiliated Xuzhou Center Hospital of Nanjing University of Chinese Medicine Xuzhou Jiangsu China; ^2^ Department of Medical College Southeast University Nanjing Jiangsu China; ^3^ Graduate School Bengbu Medical College Bengbu Anhui China; ^4^ Department of Pharmaceutical Sciences College of Pharmacy and Health Sciences St. John's University, Queens New York New York USA

**Keywords:** cell signaling, diseases, intervention therapy, protein kinases, protein phosphorylation

## Abstract

Protein phosphorylation is an important post‐transcriptional modification involving an extremely wide range of intracellular signaling transduction pathways, making it an important therapeutic target for disease intervention. At present, numerous drugs targeting protein phosphorylation have been developed for the treatment of various diseases including malignant tumors, neurological diseases, infectious diseases, and immune diseases. In this review article, we analyzed 303 small‐molecule protein phosphorylation kinase inhibitors (PKIs) registered and participated in clinical research obtained in a database named Protein Kinase Inhibitor Database (PKIDB), including 68 drugs approved by the Food and Drug Administration of the United States. Based on previous classifications of kinases, we divided these human protein phosphorylation kinases into eight groups and nearly 50 families, and delineated their main regulatory pathways, upstream and downstream targets. These groups include: protein kinase A, G, and C (AGC) and receptor guanylate cyclase (RGC) group, calmodulin‐dependent protein kinase (CaMK) group, CMGC [Cyclin‐dependent kinases (CDKs), Mitogen‐activated protein kinases (MAPKs), Glycogen synthase kinases (GSKs), and **C**dc2‐like kinases (CLKs)] group, sterile (STE)‐MAPKs group, tyrosine kinases (TK) group, tyrosine kinase‐like (TKL) group, atypical group, and other groups. Different groups and families of inhibitors stimulate or inhibit others, forming an intricate molecular signaling regulatory network. This review takes newly developed new PKIs as breakthrough point, aiming to clarify the regulatory network and relationship of each pathway, as well as their roles in disease intervention, and provide a direction for future drug development.

## Graphical abstract

Human protein phosphorylation kinases were divided into eight groups and nearly 50 families, and delineated their main regulatory pathways, upstream and downstream targets. We analyzed more than 300 small‐molecule protein phosphorylation inhibitors listed by Food and Drug Administration or in clinical research for classification, generalization, and analysis. This review takes newly developed drugs as entry point, aiming to clarify the regulatory network and relationship of each pathway, as well as their roles in disease intervention, and provide a direction for future drug development.

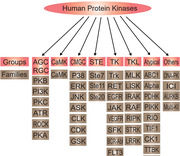

## 1 INTRODUCTION

Protein phosphorylation is a common post‐transcriptional modification and plays an important role in intracellular signaling transduction. Protein phosphorylation refers to a process of transferring the phosphate group of adenosine triphosphate (ATP) to the amino acid residues (serine, threonine, tyrosine) of the substrate protein catalyzed by protein kinase, or binding to guanosine triphosphate (GTP) under the action of a signal. Protein phosphorylation is a common regulation method in organisms, and plays an important role in the process of cell signal transduction.^1^ Protein phosphorylation is the most basic, common, and important mechanism for regulating and controlling protein activity and function. Phosphorylation of proteins can allosteric proteins, activate the corresponding protein activity, then form protein complexes with different proteins, and then further promote protein phosphorylation, which constitutes the basic mechanism of cell signal transduction.^2^ Human protein kinases constitute about 1.7% of the total genes and mediate most signaling transduction in eukaryotic cells. In eukaryotes, there are more than 100,000 phosphorylation regulatory sites, involving many functions and influences such as cancer mutation, genetic variation, mRNA expression, DNA methylation, and molecular interaction.^3^ The genetic alterations including mutation, overexpression, inhibition, and translocation are involved in cell signaling and development of diseases.

Because protein kinases are the most important components of intracellular signaling proteins, they also become important targets for drug intervention. Since the first approval of imatinib in 2001, small‐molecule kinase inhibitors have brought enormous benefits to patients in various therapeutic areas.^4^ Currently, one‐third of the new drug development is focused on targeting protein phosphorylation kinases.^4^ A database of management, annotation, and updating of protein kinase inhibitors (PKIs) in clinical trials, named Protein Kinase Inhibitor Database (PKIDB) (https://www.icoa.fr/pkidb/), provides the latest clinical trials on PKIs.^5^ Up to date, there are 303 kinds of compounds under investigation. Up to the beginning of 2022, there are 68 small‐molecule PKIs approved by the Food and Drug Administration (FDA) of the United States.^6^ Besides, there are many new types of PKIs that were under preclinical research. There are still some difficulties and challenges in the drug development process, such as high electrophilic reactivity, non‐specific cytotoxicity, and target cysteine mutation disadvantages relative to reversible kinase inhibitors that previously hindered the development of small‐molecule kinase inhibitors.^7^

This review takes the PKIs that have been clinically used and are undergoing drug clinical trials as the entry point, and introduces the signaling pathways, regulatory pathways, mechanisms of action, and regulatory sites related to different groups of phosphorylated kinases (Table S1). Human protein kinases are divided into groups, families, and subfamilies. There are eight main groups^8^: (1) protein kinase A, G, and C (AGC) and receptor guanylate cyclase (RGC) group, (2) calmodulin‐dependent protein kinase (CaMK) group, (3) CMGC [**C**yclin‐dependent kinases (CDKs), **M**itogen‐activated protein kinases (MAPKs), **G**lycogen synthase kinases (GSKs), and **C**dc2‐like kinases (CLKs)] group, (4) sterile (STE)‐MAPKs group, (5) tyrosine kinases (TK) group, (6) tyrosine kinase‐like (TKL) group, (7) atypical kinases group, and (8) other group. The characteristics, upstream and downstream regulation of PKI in different groups are introduced; PKI‐related diseases and intervention therapy are also introduced in this review.

## 2 KINASE GROUPS

### 2.1 AGC and RGC kinase group

**FIGURE 1:**
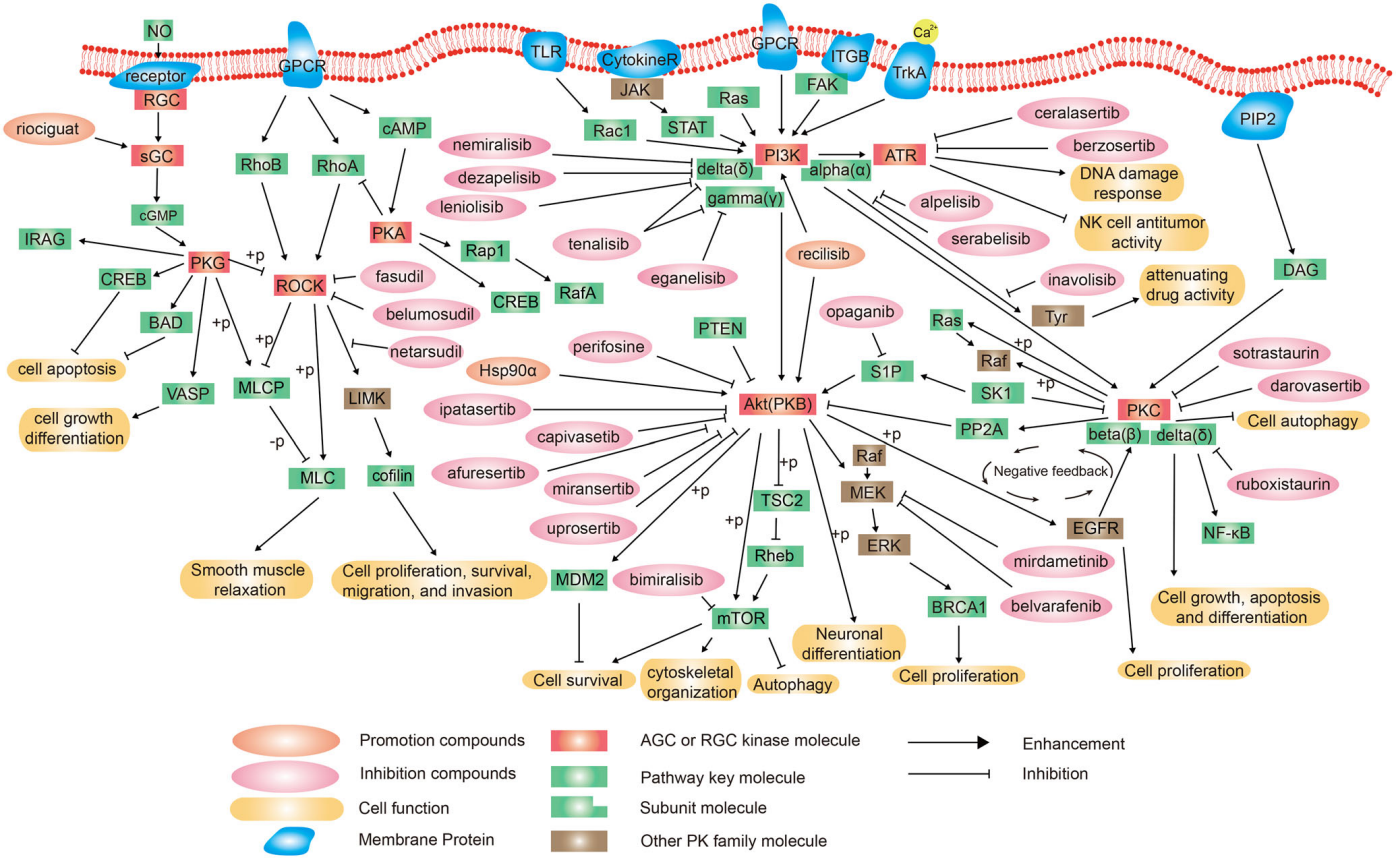
Schematic diagram of protein phosphorylation signaling pathway of protein kinase A, G, and C (AGC)‐receptor guanylate cyclase (RGC) group.

Besides targeting AKT, some inhibitors target downstream signaling pathways mediated by AKT as will be discussed. One example is the nerve growth factor (NGF), pathway that activates AKT through its receptor, the TK receptor tropomyosin‐related kinases A (TrkA)^9^ promoting neuronal survival and neurite outgrowth. The AGC kinase group consists of seven families, including protein kinase A (PKA), protein kinase B (PKB), protein kinase C (PKC), protein kinase G (PKG), phosphatidylinositol 2‐kinase (PI2K), phosphatidylinositol 3‐kinase (PI3K), rho‐associated coiled‐coil‐containing kinase (ROCK), and RGC families. The family members of the AGC kinase group have a hydrophobic motif sequence, which is composed of a universal motif, docked at the coevolutionary hydrophobic site in the core lobule of human protein kinase^10^ (Figure 1).

#### 2.1.1 PKB family

PKB, which is known as AKT, acts as a downstream molecule of the PI3K. AKT regulates many cellular processes such as cell proliferation and survival, metabolism, tumor growth, and metastasis.^11^ Neurotrophins, such as NGF, can activate methyl ethyl ketone (MEK)/extracellular signal‐regulated kinase (ERK)/AKT pathway to promote neuronal survival and neurite outgrowth by activating the TK receptor TrkA, for Ca^2+^‐dependent AKT phosphorylation.^9^

AKT is involved in a variety of cellular programs, such as cell survival‐related glucose metabolism, cell apoptosis, cell proliferation, transcription, and cell migration. Thus, AKT is a good therapeutic target for diseases related to cell proliferation, glucose metabolism, and others.

AKT phosphorylation inhibitors are most commonly used in the treatment of cancers. Phosphatidylinositol‑4,5‑bisphosphate 3‑kinase catalytic subunit alpha (PIK3CA) is associated with hemangiosarcoma (HSA) and is phosphorylated by AKT, which enhances epidermal growth factor receptor (EGFR) signaling.^12^ Alpelisib, a molecular drug targeting PIK3CA mutations, has significant antitumor affects in canine PIK3CA‐mutated HSA cell lines in animal experiments.^12^ Multiple studies have demonstrated the synergistic antitumor activity using the combination of AKT pathway inhibitors, such as capivasertib, in breast cancer susceptibility genes (BRCA)‐related cancers.^13^ Studies show that reduced AKT activity by AKT inhibitors is associated with reduced tumor cell proliferation^14^ and multidrug resistance.^15^ Several drugs of AKT inhibitor candidates such as afuresertib, ipatasertib, uprosertib, perifosine (KRX‐0401), and PHT‐427 have been studied.^14^ Opaganib and sphingosine‐1‐phosphate (S1P) regulate AKT and affect cell proliferation, migration, colony formation, and apoptosis in metastatic melanoma.^16^

AKT phosphorylation is involved in other diseases such as toluene diisocyanate (TDI)‐related asthma. Peng et al.^17^ demonstrated by an animal asthma model that inhibition of AKT by MK2006 prevents TDI‐induced airway inflammation. Besides, one orally AKT inhibitor, ARO092, attenuates neutrophil–platelet interactions in sickle cell disease.^18^ Nandan et al.^19^ demonstrated that an AKT inhibitor, miransertib (ARQ092), enhanced mammalian target of rapamycin (mTOR)‐dependent autophagy in Leishmania‐infected macrophages.

Hsp90α promotes the AKT phosphorylation signaling pathway to stimulate the repair process of corneal injury. Thus, recombinant Hsp90α is expected to be a candidate drug for the treatment of corneal injury.^20^ This exploits the pro‐proliferative effects of AKT agonists, which play an important role in the treatment of diseases requiring cell proliferation. PI3K/AKT signaling pathway activated by recilisib inhibits cardiomyocyte apoptosis during myocardial infarction.^21^

There are no drugs of AKT inhibitor that are approved by the FDA,^6^ but four drugs (afuresertib, capivasertib, ipatasertib, and uprosertib) are under clinical trials that can be found in the PKIDB.^5^ These drugs are used in the treatment of spindle cell/sclerosing rhabdomyosarcoma and other diseases because of their good signal blocking and cell growth inhibitory activity.^22^

#### 2.1.2 PI3K family

PI3K is the upstream molecule of PI3K/AKT signaling pathway and has the same biological function as AKT. PI3K has three subunits including PI3Kα, PI3Kδ, and PI3Kγ.

PI3Kδ is a lipid kinase that phosphorylates the D‐3 position of the phosphatidylinositol ring.^23^ The new highly selective PI3Kδ inhibitor, INCB050465 (Parsaclisib), was found to be a good drug with an excellent profile through in vivo pharmacodynamic and efficacy studies in animal experiments.^23^ Other PI3Kδ inhibitors (leniolisib and seletalisib) were used for the therapy of the activated phosphoinositide 3‐kinase delta syndrome (APDS).^24^ It has been demonstrated that an inhaled nemiralisib, a PI3Kδ inhibitor, was effective and safe for the treatment of chronic obstructive pulmonary disease (COPD).^25^

PI3Kγ is another subunit and can be inhibited by eganelisib, which inhibits cell proliferation, self‐renewal, migration, and invasion in vitro.^26^ There were some dual PI3K δ/γ inhibitors, for example tenalisib, whose phase I clinical trial was completed with acceptable safety up to 1200 mg twice daily with no dose limiting toxicity.^27^ It is promising to become an important PI3K inhibitory antitumor drug, used for the treatment of many cancer patients such as relapsed/refractory T‐cell lymphoma.^28^

However, several studies have shown that some PI3K pathway inhibition can negatively feedback activate TK receptor signaling, which then re‐blocks the pathway and reduces the therapeutic activity of the drug.^29^ This is related to PI3Kα (encoded by PIK3CA), a p110α catalytic subunit,^30^ which is inhibited by compounds such as alpelisib and serabelisib.^31^

In the PKIDB or FDA approved drugs, there are nine drugs targeting PI3Kδ inhibition: dezapelisib, leniolisib, nemiralisib, parsaclisib, puquitinib, samotolisib, seletalisib, tenalisib, and umbralisib that are under clinical trials.^5^, ^6^ These drugs are used in the treatment of various diseases, including immunologic deficiency syndromes, disease susceptibility, genetic predisposition to disease, Epstein–Barr virus infections, COPD, asthma, eosinophilia, pneumonia, and respiratory tract diseases.^25^

#### 2.1.3 PKC family

PKC is an intracellular receptor for phorbol esters with tumor‐promoting activity and is considered a key player in carcinogenesis.^32^ Studies have shown that the activation of PKC is essential to induce cell differentiation, proliferation, metastasis, and survival.^33^ However, there are some studies which show that PKC also plays a role as a tumor suppressor, and suggest that future clinical work is likely to focus on restoring rather than inhibiting PKC pathway activity.^34^

In the PKIDB or FDA approved drugs, there are four drugs targeting PKC inhibition^5^, ^6^: ruboxistaurin, enzastaurin, sotrastaurin (AEB071), and darovasertib (IDE196). Ruboxistaurin is a PKCβ inhibitor, which has an activity for the treatment of diabetes, hypertension, kidney failure, and osteoarthritis.^35^ Sotrastaurin is suggested to be involved in cytopathogenic effect and lymphocyte activation, and is used for the treatment of cancers such as uveal neoplasms, melanoma, urinary bladder neoplasms, esophageal neoplasms, and other diseases like cartilage diseases, insulin resistance, and diabetes mellitus.^36^, ^37^ Darovasertib is under a phase Ib clinical trial for uveal neoplasms treatment.^38^

#### 2.1.4 Ataxia telangiectasia and Rad3‐related family

Ataxia telangiectasia and Rad3‐related (ATR) kinase is one of the major kinases in the DNA damage response signaling pathway, which responds to replication stress as well as DNA damage caused by various genotoxic factors.^39^ Many ATR inhibitors, such as ceralasertib, are reported to combining the inhibitory effect of DNA damage response and the effect on immune checkpoint blockade to boost antitumor activity of NK cells.^40^ Berzosertib, an ATR inhibitor, is found to have an effect on cancer therapy and is settled in a multicenter, open‐label, randomized, phase II trial.^41^ It has been used for the treatment of lung neoplasms, esophageal neoplasms, ovarian neoplasms, and osteosarcoma in the clinical trials.^42^, ^43^

#### 2.1.5 ROCK family

ROCK can promote actin‐myosin‐mediated contractility through the phosphorylation process of various proteins. ROCK plays an important role in cell proliferation, differentiation, apoptosis, and oncogenic transformation.^10^ They regulate many key cellular functions, including proliferation, exercise, and vitality. RhoA/ROCK signaling has been shown to be closely related to arterial hypertension, cardiovascular and renal remodeling, hypertensive nephropathy, and post‐transplant hypertension.^44^

There are four drugs targeting ROCK in the PKIDB or FDA approved drugs: belumosudil, fasudil, netarsudil, and ripasudil.^5^, ^6^ Belumosudil (KD025) is a ROCK2 inhibitor, which modulates Th17/regulatory T‐cell balance and controls profibrotic pathways, which offers a novel approach for the treatment of chronic graft‐versus‐host disease (cGVHD) and systemic sclerosis.^45^, ^46^ Fasudil is an inhaled ROCK inhibitor, which affects the cascade of reactions involving various proinflammatory cytokine molecules, mainly for the treatment of cardiovascular diseases.^47^ The clinical use of netasudil is limited to patients with glaucoma or ocular hypertension, and netasudil has a potential to affect Lin11, Isl‐1, and Mec‐3 kinase (LIMK) pathway for cancer treatment.^48^ Ripasudil is used in glaucoma, ocular hypertension, blepharitis, conjunctival diseases macular edema, cataract, and retinopathy.^49^ Additionally, ROCK can be considered as a key therapeutic target for the treatment of COVID‐19.^50^

#### 2.1.6 PKA family

PKA is regulated by cAMP and affects downstream cAMP response element‐binding (CREB) protein, forming the cAMP‐PKA‐CREB signaling pathway. It is known that the cAMP‐PKA‐CREB signaling pathway is related to osteoblasts, beta2‐adrenergic response, cell apoptosis, central nervous system (CNS) functional recovery, and upregulation of nuclear factor‐κB (NF‐κB).^51^ It is demonstrated that some Chinese medicines, such as Zuogui Pill and Yougui Pill, regulate the PKA signaling pathway by improve cAMP levels and PKA expression.^52^ Besides, it is demonstrated that liraglutide, a PKA inhibitor, has an effect on the mesenchymal stem cells for acute lung injury and acute respiratory distress syndrome.^53^ But there are few drugs targeting PKA, most likely due their wide distribution in cells.

#### 2.1.7 Receptor guanylate cyclase family

Two types of guanylate cyclase (GC) exist in mammals, which are distinguished by their location within the cell. Membrane‐bound guanylate cyclase (mGC) is the first type of GC‐coupled receptor.^15^ The natriuretic peptides (NP) activate mGC. Seven subtypes of transmembrane mGC have been identified (GC‐A to GC‐G) in mice.^54^ GC‐A has beneficial properties such as regulating blood pressure and natriuretic, which is activated by two types of NP: ANP and BNP. At present, GC‐A has become an essential target for treating cardiovascular diseases.^55^ CNP activates GC‐B. In failing hearts, the CNP inhibits cardiac remodeling by activating the homologous receptor, GC‐B.^56^ Intracellular soluble guanylate cyclase (sGC) is the second type of GC. In physiological processes, nitric oxide (NO) is diffused into the vessel lumen and vessel wall, activating sGC, which functions through the NO‐sGC‐cGMP signaling pathway. The NO‐sGC‐cGMP pathway is an essential regulator of myocardial function and vascular tone.^57^

Since preclinical and clinical trials have been developed. GC has been viewed as an attractive target for pharmacological intervention. Riociguat is a stimulant of GC, which leads to relaxation of vascular smooth muscle. At present, riociguat has been approved for the treatment of two kinds of pulmonary hypertension [pulmonary arterial hypertension (PAH)/chronic thromboembolic pulmonary hypertension (CTEPH)].^58^ Versiciguat is a sGC stimulator that stimulates the production of cGMP and sGC independent of NO and enhances the effects of NO by stabilizing its binding to sGC. Versiciguat was recently approved in the USA to reduce the risk of heart failure in those with a 45% ejection fraction.^59^ Praliciguat and olinciguat are newly developed sGC stimulators for the treatment of chronic heart failure.^59^, ^60^ Since severe renal diseases are mostly due to downregulation of NO‐sGC‐cGMP signal transduction, the current phase II and III clinical trials for vericiguat, praliciguat, and olinciguat are undertaken for the treatment of kidney diseases.^61^, ^62^, ^63^ There is increasing evidence showing that this medication can effectively treat heart failure, kidney disease, and pulmonary hypertension and can be used as first‐line therapies.

### 2.2 CaMK group

**FIGURE 2:**
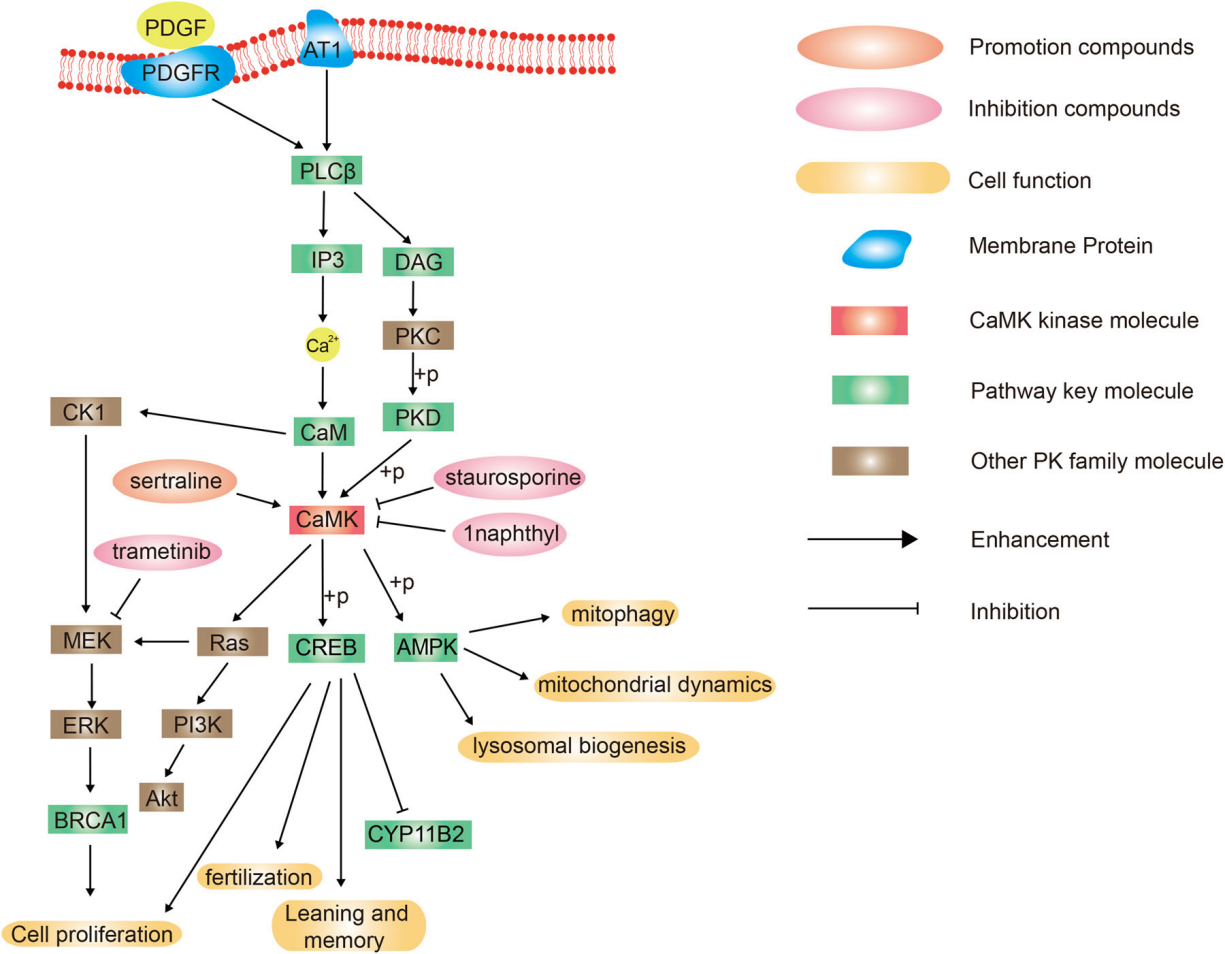
Schematic diagram of protein phosphorylation signaling pathway of calmodulin‐dependent protein kinase (CaMK) group.

CaMK group has only one family member. CaMK II, activated by the Ca^2+^/calmodulin complex, is a serine/threonine kinase of the CaMK group.^8^ CaMK II responses to neurohormonal stimulation, promotes cardiac remodeling, and increases the risk of arrhythmia.^64^ CaMK is regulated by the diacylglycerol (DAG)/PKC/PKD pathway. It regulates Ras, leading to the activation of the PI3K/AKT, as well as the MEK/ERK signaling pathways.^65^ CaMK‐dependent activation of adenosine monophosphate (AMP)‐activated protein kinase (AMPK) is involved in mitotic phagocytosis, mitochondrial dynamics, and lysosomal biogenesis in microglia, which in turn produce cytotoxicity caused by mitotic phagocytosis and cathepsin B activation^66^ (Figure 2).

Some CaMK inhibitors, such as staurosporine and 1naphthyl PP1, were under investigation for the treatment of schistosomiasis.^67^ However, there is no CaMK inhibitor in a clinical trial in the PKIDB.

### 2.3 CMGC group

**FIGURE 3:**
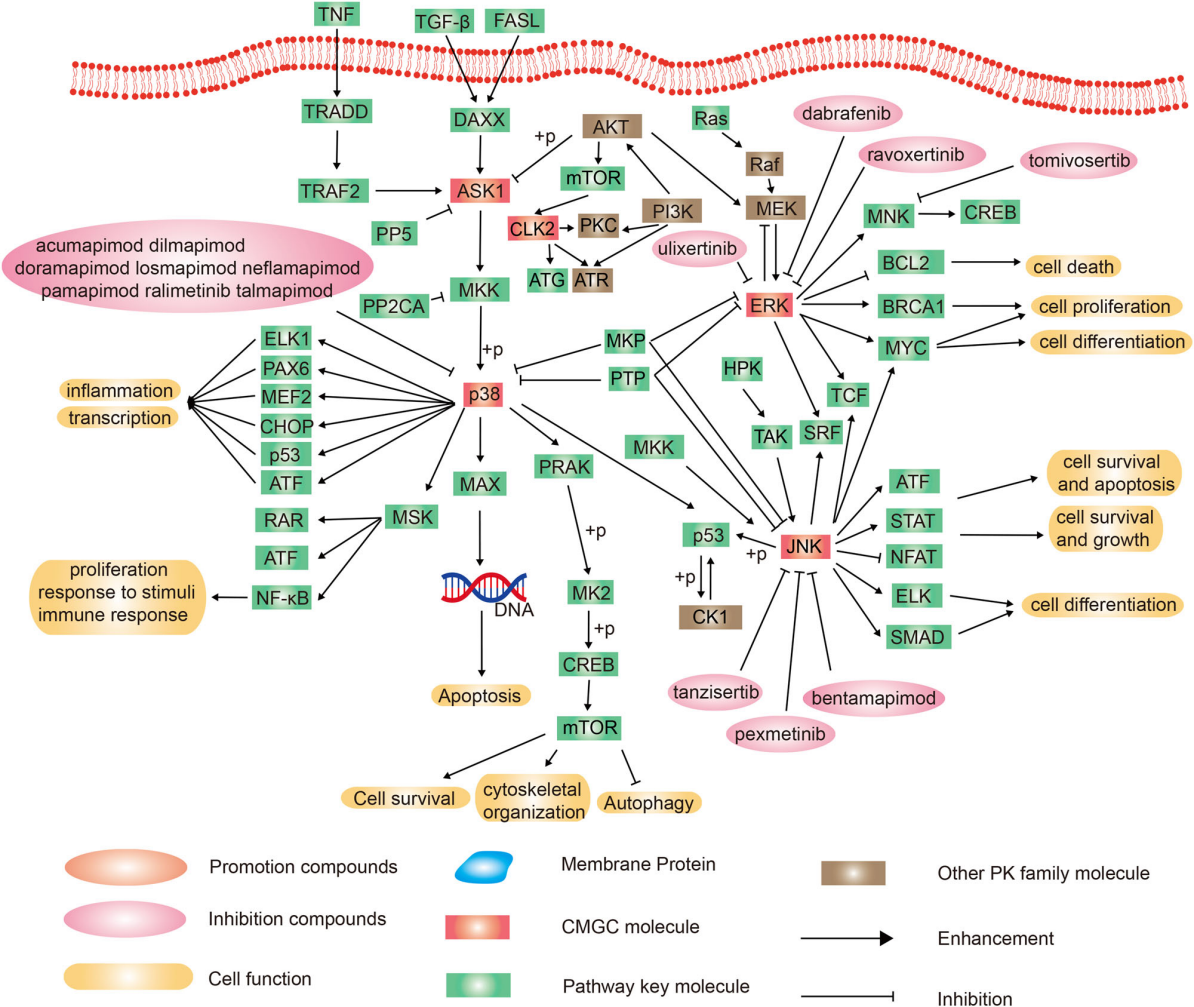
Schematic diagram of protein phosphorylation signaling pathway of the extracellular signal‐regulated kinase (ERK), P38, c‐Jun N‐terminal kinase (JNK), apoptosis signal‐regulating kinase (ASK), and Cdc2‐like kinase 2 (CLK2) families in CMGC (**c**yclin‐dependent kinases, **m**itogen‐activated protein kinases, **g**lycogen synthase kinases, and **C**dc2‐like kinases) group.

CMGC group contains seven main families including p38 mitogen‐activated protein kinases (p38 MAPKs), ERKs, c‐Jun N‐terminal kinases (JNKs), apoptosis signal‐regulating kinase 1 (ASK1), CLK2, CDKs, and GSK3. There are 31 drugs targeting the CMGC group in the PKIDB or in FDA approved drugs: eight in p38 MAPKs family, three in JNK family, five in ERK family, and 14 in CDKs family^5^ (Figure 3).

#### 2.3.1 p38 MAPKs family

The p38 MAPK is an important signaling molecule, which responds to environmental and intracellular stress, and is related to cell proliferation, apoptosis, inflammation, metastasis, and angiogenesis (Figure 3).^68^, ^69^

There are eight drugs targeting the p38 MAPK family in the PKIDB or in FDA approved drugs^5^: acumapimod, dilmapimod, doramapimod, losmapimod, neflamapimod, pamapimod, ralimetinib, and talmapimod. These p38 MAPK inhibitors are often used in various inflammatory diseases. Acumapimod is an oral p38 MAPK inhibitor, which inhibits p38 MAPK‐regulated inflammatory response and is used for the treatment of COPD.^70^ Dilmapimod is used for the prevention of acute respiratory distress syndrome and other organ damage in some large clinical trials.^71^ BIRB796 (doramapimod), another p38 MAPK inhibitor, is used for the treatment of inflammatory bowel diseases.^72^ In addition to their therapeutic effects in inflammatory diseases, some p38 MAPK inhibitors, such as neflamapimod, are used in the improvement of cognition and walking disorders.^73^ Additionally, p38 MAPK is considered as a key therapeutic target for the treatment of COVID‐19.^50^

#### 2.3.2 ERKs family

ERK regulates the transcriptional activation of Elk‐1, activating transcription factor (ATF), Ap‐1, c‐fos, and c‐Jun in cells, and participates in various biological responses such as cell proliferation and differentiation, morphological maintenance, skeleton construction, apoptosis, and carcinogenesis.^74^, ^75^ ERKs are the key protein kinase nodes involved in Ras‐Raf‐MEK‐ERK‐MAP kinase signaling pathway, and involved in many biological processes including cell proliferation and apoptosis, immune response, nervous system function, and RNA synthesis and processing.^76^

There are four drugs targeting ERKs family in the PKIDB or in FDA approved drugs^5^: dabrafenib, ravoxertinib, tomivosertib, and ulixertinib. Dabrafenib is widely used in the treatment of melanoma, skin neoplasms, brain neoplasms, lung cancer, thyroid cancer, and others because of its ERK inhibitory effect.^77^, ^78^ It is reported that IL4/IL4R signaling can activate the ERK pathway, affect osteoclast, and lead to osteolytic lesions, and can be inhibited by ravoxertinib.^79^ Tomivoltinib is a highly potent and highly selective inhibitor of MNK1/2, which inhibits MNK‐eIF4E‐β‐catenin and increases the sensitivity of gastric cancer to chemotherapy.^80^

#### 2.3.3 JNKs family

JNK, also known as stress‐activated protein kinase (SAPK), plays an important role in a variety of biological and pathological processes including cell cycle, apoptosis infection, reproduction, and cellular stress.^81^, ^82^

There are three drugs targeting JNKs family in the PKIDB or in FDA approved drugs^5^: bentamapimod, pexmetinib, and tanzisertib. Bentamapimod is a specific c‐JNK inhibitor, which increases the sensitivity of many tumors to chemotherapeutic drugs.^83^ For example, it increases the sensitivity of A2780 ovarian stem cells to carboplatin and paclitaxel,^84^ and sensitizes glioma cells to temozolomide and vincristine.^85^ Another JNKs inhibitor, pexmetinib, is used for the treatment of myelodysplastic syndromes and acute myeloid leukemia (AML).^86^ Tanzisertib is a newly discovered c‐JNK inhibitor. It is related in fibrosis and is in clinical trials for the treatment of idiopathic pulmonary fibrosis.^87^

#### 2.3.4 ASK1 family

ASK1 kinase is the upstream molecule of p38 kinase and JNK kinase families.^88^ It is regulated by tumor necrosis factor (TNF), transforming growth factor‐β (TGF‐β), Fas Ligand [FAS (Fas Cell Surface Death Receptor) is a protein coding gene's name] and more, and inhibited by the AKT kinase family.^89^ ASK1 is a crucial cellular stress sensor, modulates diverse responses to oxidative and endoplasmic reticulum stress and calcium influx.^90^ Although there are many drugs targeting its downstream p38 and JNK family, adverse reactions with serious side effects were observed. Therefore, inhibitors targeting ASK1 were proposed by many scholars to better the therapeutic effects.^90^

Currently, inhibiting the ASK1 kinase family is expected to intervene diseases such as neoplasms, non‐alcoholic fatty liver disease, cerebral ischemia, Parkinson's disease, insulin resistance, diabetes, Alzheimer's disease, melanoma, and more. At present, no drug that specifically inhibits the ASK1 kinase family has been approved by the FDA, and no ASK1 kinase inhibitor has participated in human clinical trials.^5^ Instead, some drugs, such as AKEX0011^88^ and GS‐444217^91^ are under basic experiments and have not yet received safety evaluation and clinical evaluation. Some scholars have also explored specific targeted inhibitory drugs through molecular docking and molecular dynamics.^92^

#### 2.3.5 CLK2 family

The CLK2 kinase family is regulated by the AKT and PI3K kinase families, and controls the ATR and the PKC families.^93^ CLK2 is closely associated with many neurodegenerative diseases, metabolic diseases, and viral infection‐related diseases, and is considered as a potential drug target for these diseases.^94^ Its specific inhibitory drugs are used in the experimental treatment of Alzheimer's disease, insulin resistance, and neoplasms, especially leukemia and triple‐negative breast cancer.^95^, ^96^ It is reported that the first CLK2 inhibitor, lorecivivint, has entered phase III clinical trials in recent years.^94^ We look forward to the therapeutic prospects of CLK2 inhibitors.

#### 2.3.6 CDKs family

The discovery of the CDKs and its regulation on cell cycle progression plays a key role in the treatment of malignant cancer.^97^ CDKs are serine/threonine protein kinases that bind to proline and play a crucial role in controlling cell cycle progression in different aspects of transcriptional regulation.^98^ Since the first report of using CDK4/6 inhibitors against cancer in 1990s, there have been thousands of reports on CDK inhibitors. CDK9 is a target enzyme for cancer therapy, but has limitations because of its adverse effects.^99^

**FIGURE 4:**
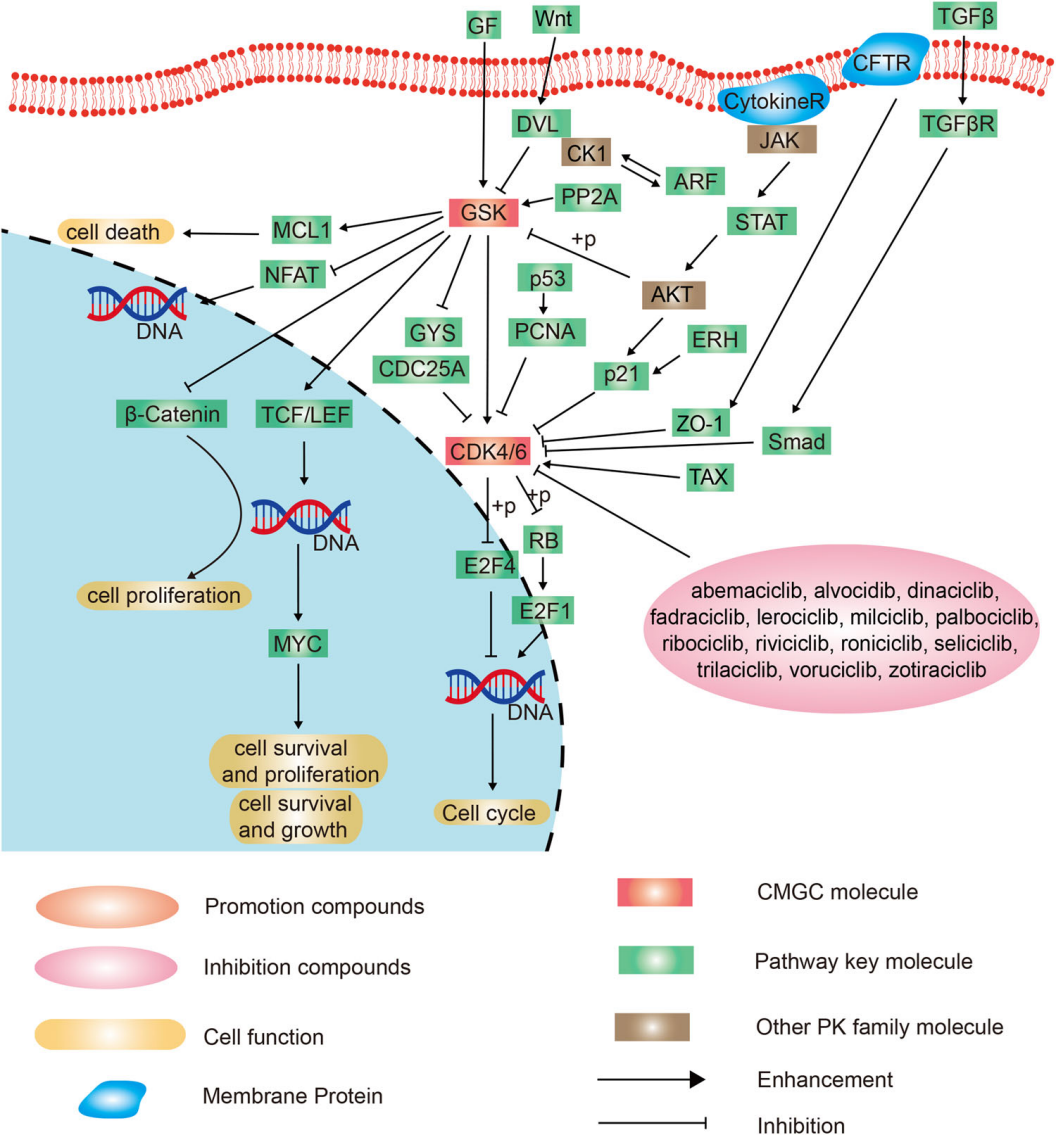
Schematic diagram of protein phosphorylation signaling pathway of the cyclin‐dependent kinases (CDKs) family in CMGC (**c**yclin‐dependent kinases, **m**itogen‐activated protein kinases, **g**lycogen synthase kinases, and **C**dc2‐like kinases) group.

There are 17 drugs targeting CDKs family in the PKIDB or in FDA approved drugs^5^: abemaciclib, alvocidib, palbociclib, ribociclib, dinaciclib, fadraciclib, lerociclib, milciclib, riviciclib, trilaciclib, voruciclib, roniciclib, seliciclib, and zotiraciclib. Abemaciclib, palbociclib, and ribociclib were the first FDA approved CDKs inhibitors. They are mainly used for combined endocrine therapy for HR+/HER2^54^ advanced or metastatic breast cancer in premenopausal/perimenopausal and postmenopausal women.^100^ Alvocidib, as well as dinaciclib, seliciclib, SNS‐032, and RGB‐286638 are CDK9 targeted inhibitors, and are mainly used for the treatment of leukemia for reversing the progression of cytarabine resistance^101^, ^102^ (Figure 4).

#### 2.3.7 GSK3 family

GSK3 is an evolutionarily highly conserved serine/threonine kinase, which participates in multiple signaling pathways and has more than 100 substrates.^103^ GSK3 regulates signaling proteins, structural proteins, and transcription factors, and participates in the regulation of cell differentiation, proliferation, survival, and apoptosis.^104^ Current research on GSK3 inhibitors is mainly focused on Alzheimer's disease. GSK3 is one of the key players in signaling pathways for normal brain function and a key molecular link between β‐amyloid (Aβ) and tau neurofibrillary tangles (NFTs).^105^ Although GSK3 was considered a good target for drug intervention nearly 10 years ago, so far, no specific GSK3 inhibitor has been certified by the FDA. The main reason may be the side effects induced by GSK3.^106^ Multiple clinical trials on GSK3 inhibitors are ongoing.

### 2.4 STE‐MAPKs group

#### 2.4.1 Ste7/MAP2K family

The mitogen‐activated protein kinase kinase (MAPKK) is a classic molecule for signal transmission from cytoplasm to nucleus. Ste7 belongs to the MAPKK family of enzymes found in yeast, which participates in pheromone signaling and nutrient deprivation/invasive growth pathways.^107^ Recent studies suggest the dual specificity of MAP2K in influencing the process of phosphorylation.^108^, ^109^

The MAPKKKs A‐Raf, B‐Raf, and C‐Raf (Raf‐1), the MAPKKs MEK1 and MEK2, and the MAPKs ERK1 and ERK2 constitute the mammalian ERK1/2 module. Signaling through the ERK pathway contributes to many cellular processes, including proliferation, differentiation, development, learning, and survival.^110^ ERK1 and ERK2 can be activated by growth factors, such as fibroblast growth factor 2 (FGF2), which causes endometriosis by regulating the ERK signaling pathway^111^; and vascular endothelial growth factor‐C (VEGF‐C), which promotes human mesenchymal stem cell migration via an ERK signaling pathway.^112^ ERK is phosphorylated and activated by MEK. Given the role of the ERK pathway in cell proliferation and diseases, some MEK inhibitors have been developed and applied in the clinic. In 2015, both the FDA and European Medicines Agency approved cobometinib, a potent, selective oral MEK1/2 inhibitor that can be taken with the B‐rapidly accelerated fibrosarcoma (BRAF) inhibitor vemurafenib for the treatment of malignant melanoma.^113^ Pimasertib is a selective MEK1/2 inhibitor in the stage of clinical experiment and shows good antitumor activity in melanoma and liver cancer.^114^, ^115^, ^116^ Refametinib and selumetinib are in clinical trials and can be found in the PKIDB for the treatment of many cancers such as hepatocellular cancer, pediatric low‐grade glioma, non‐small‐cell lung cancer (NSCLC), and melanoma.^117^, ^118^ There are two kinds of MEK inhibitors, belvarafenib and mirdametinib that were under clinical trials for the treatment of malignant tumors such as melanoma.^14^

#### 2.4.2 Ste11/MAP3Ks family

Ste11/MAP3Ks are protein Ser/Thr kinases activated by extracellular stimuli through phosphorylation.^119^ MAP3Ks phosphorylate both Ser/Thr residues of MAP2Ks’ activation loops. By phosphorylating signaling effectors and interacting specifically with proteins, the MAP3Ks activate MAP2K‐MAPK pathways in response to stimuli.^120^

Rapidly accelerated fibrosarcoma (RAF) is a subfamily of MAP3Ks and a member of cytoplasmic serine/threonine kinases. RAF contributes to oncogenesis and critical physiological processes, including cell growth. The expression of C‐Raf is widespread, but that of A‐Raf and B‐Raf is limited to selected tissues. A‐Raf is primarily found in urogenital and gastrointestinal tissues. B‐Raf is mainly found in neural, testicular, splenic, and hematopoietic tissues. Raf molecules have distinct physiological functions in the development and progression of diseases, as shown by their expression patterns.^121^ MEK/ERK pathway is activated when A‐Raf is active. The A‐Raf protein interacts with proteins located in different cellular compartments to control apoptosis, RNA catabolism, and GTPase activity.^122^ Clinically, dominant‐activating mutations in B‐Raf have been associated with melanoma, colorectal cancer, breast cancer, and glioma.^123^, ^124^ C‐Raf represents the prototypical Raf family member, and is widely expressed. When C‐Raf is active, it activates MEK/ERK, resulting in increased resistance to apoptosis mediated by B‐cell CLL/Lymphoma 2 in the heart and liver cells.^125^ More and more Raf kinase inhibitors have been developed and applied. Patients with metastatic colorectal cancer carrying the B‐Raf V600E mutation are benefited from a combination therapy of encorafenib, cetuximab, and binimetinib with significantly longer overall survival.^126^ It is more effective to use MEK1/2 inhibitors in conjunction with B‐Raf inhibitors in treating melanoma, which has become the standard treatment. FDA approved the combinations of vemurafenib and cobimetanib, dabrafenib and trametinib, and encorafenib plus binimetinib for the treatment of BRAFV600E melanoma.^127^

#### 2.4.3 Ste20/MAP4K family

The MAP4Ks are members of the Ste20‐like family of serine/threonine kinases in mammals. MAP4Ks have been reported to activate JNK by activating the cascade of MAP3K‐MAP2K, including MAP4K1/hematopoietic progenitor kinase 1 (HPK1), MAP4K2/GCK, MAP4K3/GLK, MAP4K4/HGK, MAP4K5/KHS, and MAP4K6/MINK.^128^, ^129^

There has been evidence showing that several MAP4Ks are essential for human disease. Autoimmune diseases may occur by downregulating HPK1 or overexpressing GLK in T cells.^130^ Type 2 diabetes and insulin resistance could result from the downregulation of HGK in T cells. The HPK1, GLK, and HGK genes play an essential role in tumor formation.^131^ These findings suggest that MAP4Ks could be used as biomarkers and drug targets to treat these diseases.

**FIGURE 5:**
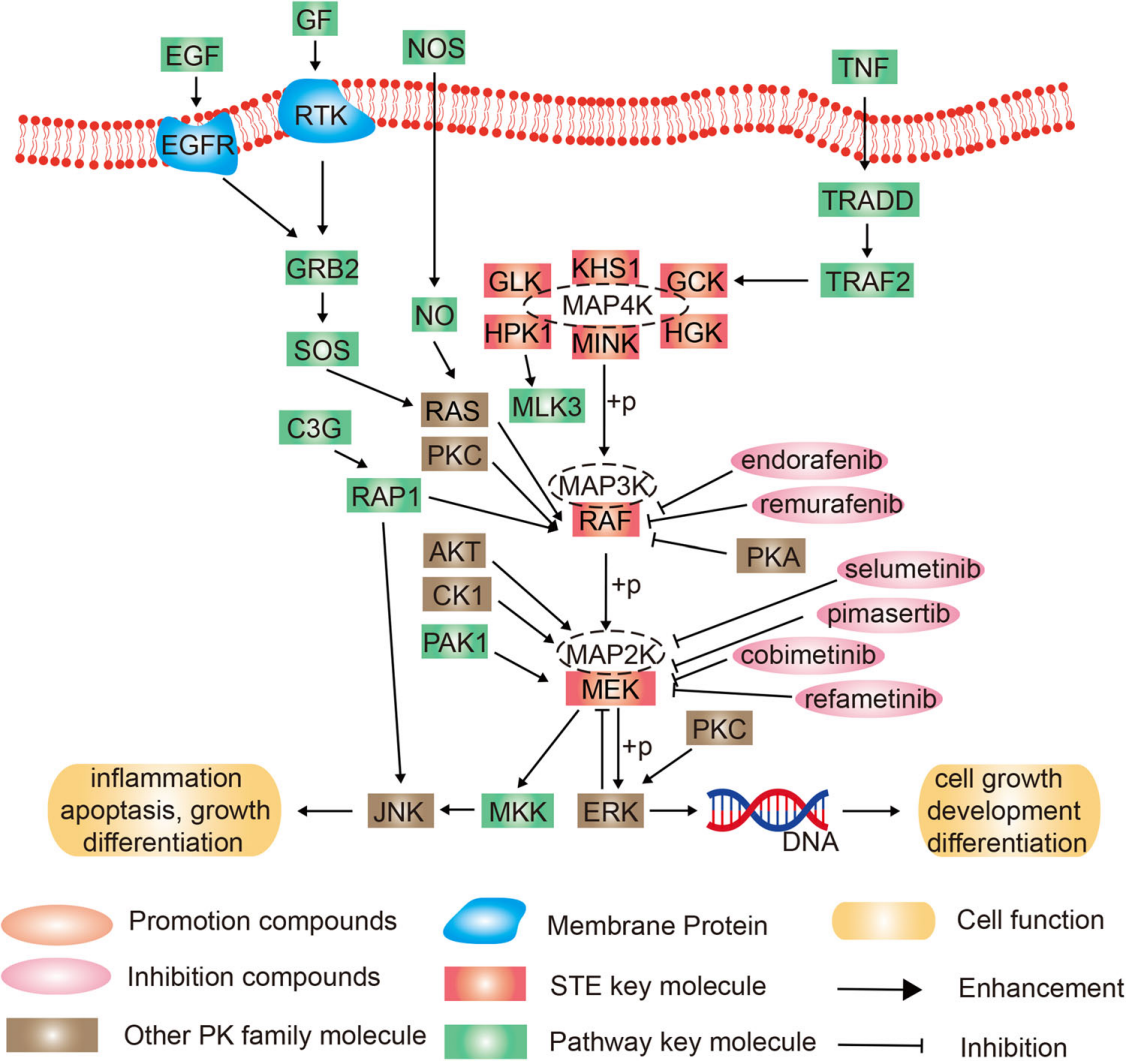
Schematic diagram of protein phosphorylation signaling pathway of STE group.

The HPK1 inhibitor (CompK/Compound 1) inhibits the signaling pathways governing T‐cell receptors (TCRs) and B‐cell receptors through phosphorylation and ubiquitination of SLP‐76 and BLK, respectively.^132^, ^133^ It is suggested that blocking HPK1 kinase activity with inhibitors alone or with checkpoint inhibition may be a practical immunotherapy approach for cancer. HPK1 deficiency subverts the inhibition of antitumor immune responses and is associated with functional augmentation of antitumor T cells. By examining how MAP4Ks functioned in different types of cells, we may be able to understand human diseases and guide clinical diagnosis and treatment (Figure 5).

### 2.5 Tyrosine kinases group

**FIGURE 6:**
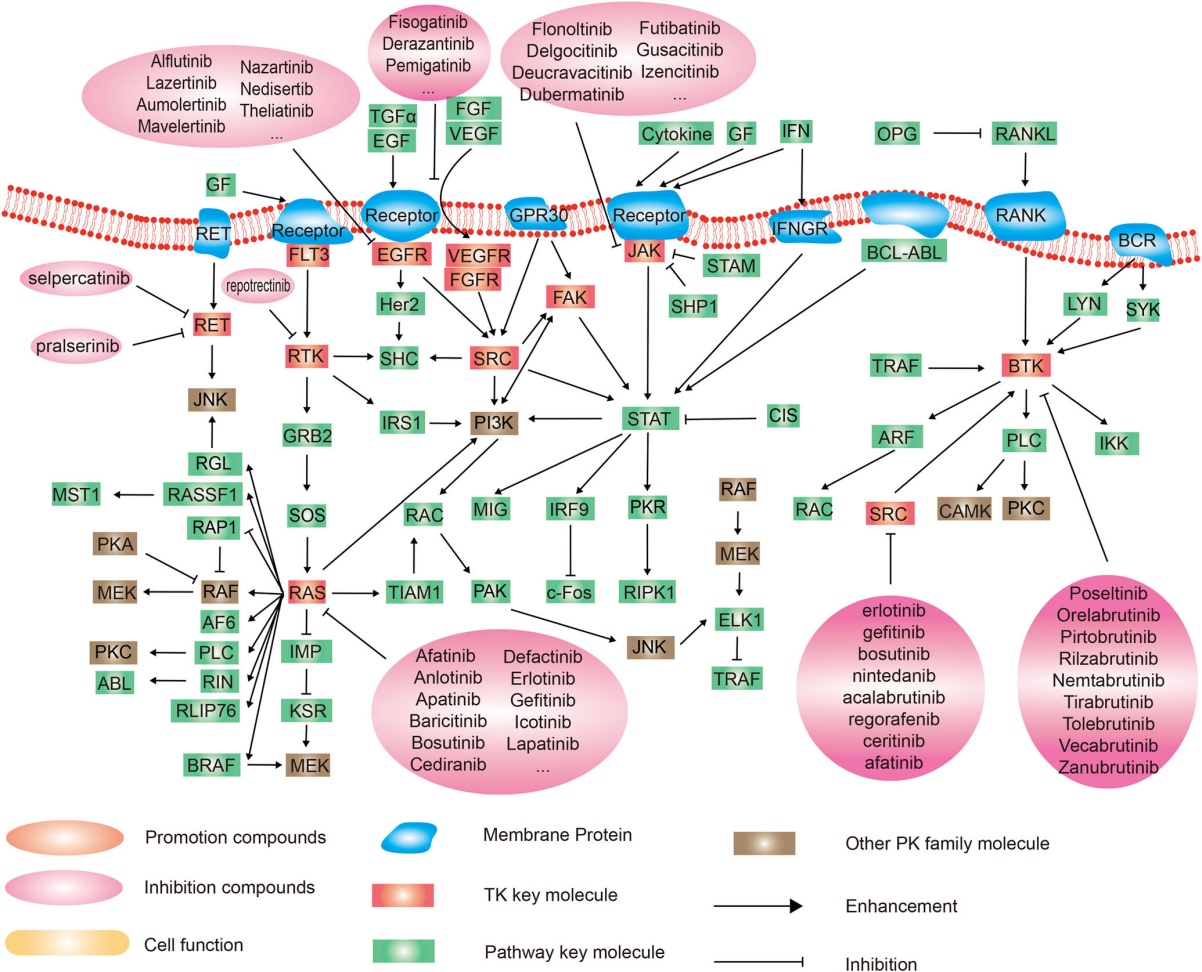
Schematic diagram of protein phosphorylation signaling pathway of tyrosine kinases (TK) group.

TKs can either be membrane bound coupled to receptors, usually growth factor receptors (RTK) or not (protein tyrosine kinase structure and function). Receptor tyrosine kinases (RTKs) are transmembrane glycoproteins that are activated by binding to their cognate ligands and relay extracellular signals to the cytoplasm by phosphorylating tyrosine residues on the receptor itself and downstream signaling proteins. The RTK family includes receptors for insulin and many growth factors, such as Trk, EGFR, FGF receptor (FGFR), VEGF, and feline mcdonough sarcoma (FMS)‐like tyrosine kinase 3 receptor (FLT3). In addition to RTKs, a large family of non‐receptor tyrosine kinases (NRTKs) exists, which includes Src‐family protein tyrosine kinases (SFK), Janus kinase (JAKs), and breakpoint cluster region‐Abelson fusion protein (BCR‐Abl), Bruton's tyrosine kinase (BTK) among others. NRTKs are components of a signaling cascade triggered by RTKs and other cell surface receptors, such as G‐protein‐coupled receptors and immune system receptors. The specific reactions catalyzed by protein tyrosine kinases are the transfer of ATP of γ‐phosphate to the hydroxyl group of tyrosine in the protein substrate (Figure 6).

#### 2.5.1 Trk family

The RTK family consists of Trks, and is present in three Trk subtypes: TrkA, TrkB as well as TrkC.^134^ Every Trk subtype possesses a significant neurotrophic ligand. TrkA is mainly triggered by NGF,^135^ and TrkB is more inclined to brain‐derived neurotrophic factor.^136^ TrkC has a preference for neurotrophin‐3 (NT‐3).^137^ Binding of specific neurotrophic ligands and their receptors can activate different downstream pathways such as PI3K, Ras/MAPK, and PLCγ, thereby promoting neuronal survival and cell differentiation. Pegcantratinib is a potent TRKA inhibitor with anti‐TRKB and TRKC activity. It is in the phase IIb clinical stage for treating inflammatory skin diseases, such as atopic dermatitis and psoriasis.^138^ Next‐generation TRK inhibitors, such as selitrectinib, repotrectinib, and taletrectinib, are in early clinical stages and available in ongoing clinical trials. They have a compact macrocyclic structure that can bind to the ATP vesicle without being hindered by the space for TRK substitution. These drugs exhibited in vitro and in vivo resistance to NTRK wild‐type and mutant kinases. These inhibitors could help patients with solid tumors resistant to existing TRK inhibitors.^139^

#### 2.5.2 RET family

Rearranged during transfection (RET) is a TK receptor.^140^ Typically, it interacts with subcellular ligands and exerts important effects in a multitude of cellular processes. RET is critical in the evolution of the central and peripheral nervous system. Mutations that activate or inhibit RET cause several aggressive illnesses, namely Hirschsprung disease and cancer. Ligand‐induced RET dimerization causes autophosphorylation of a wide range of tyrosine residues and contributes to the activation of intracellular signaling cascades, which impacts many cellular processes.^141^ RET inhibitors, selpercatinib and pralsetinib, are in phase III clinical trials and approved for the therapeutic use in individuals with metastatic RET fusion‐positive NSCLC by comparing them with standard first‐line therapy to observe its efficacy in RET‐positive NSCLC patients.^142^

#### 2.5.3 EGFR family

EGFR is a TK, which participates in cellular processes and cancer progression.^143^ EGFR, a classical transmembrane receptor, activates multiple downstream effectors and TKs by means of ligand‐induced dimerization, triggering a signaling cascade response. EGFR‐activated phosphorylation responses promote signaling cascades and are associated with embryogenesis and stem cell division.^144^ The third‐generation EGFR‐tyrosine kinase inhibitors (TKI) that target T790M mutations include alflutinib, nazartinib, lazertinib, and more. Alflutinib is a newly developed third‐generation EGFR‐TKI that is currently in phase I/II studies.^144^ Alflutinib is currently found to have clinical efficacy and acceptable toxicities in patients with advanced NSCLC with EGFR T790M mutations, including those with CNS metastases. Safety, clinical activity, and pharmacokinetics of alflutinib (AST2818) in patients with advanced NSCLC with EGFR T790M mutation. Nazatinib is in phase I/II and has been found to have a high safety profile with low skin toxicity and adverse effects such as maculopapular rash.^145^ Lazertinib is used for the treatment of NSCLC. Lazertinib was approved for the first time for the treatment of EGFR T790M mutation‐positive patients with locally advanced or metastatic NSCLC who had previously received EGFR‐TKI therapy.^146^

#### 2.5.4 JAK family

The JAK family of TKs includes JAK1, JAK2, JAK3, and tyk2.^147^ The JAK phosphorylates signal transducers and activators of transcription (STAT), dimerizes, and then translocates through the nuclear membrane to the nucleus to mediate the manifestation of the pertinent genes. This can be referred to as the JAK/STAT signaling pathway. In recent years, cytokine‐stimulated signaling pathway, known as the interleukin (IL)‐6 signaling pathway, was found.^148^ The signaling pathway is associated with all kinds of body functions and is participating in a number of important biological processes, including cell proliferation, differentiation, apoptosis, and immune regulation.^149^ At present, research on the JAK/STAT pathway associated with disease and drug innovation is concentrated in inflammatory and neoplastic diseases. JAK inhibitors as next‐generation targeted therapies for atopic dermatitis, such as delgocitinib, are used to treat atopic dermatitis in adults and children in Japan, but their safety, durability, and effectiveness still require extensive clinical trials.^150^

#### 2.5.5 VEGFR family

Due to the wide variety of TKs, among which VEGF, one of the main pro‐angiogenic factors, performing its function mostly by stimulating two TK receptors. Numerous reported studies have drawn attention to the critical role of VEGFR‐2 in tumorigenesis and tumor progression.^151^ VEGFR‐2 activation is associated with phosphorylation of multiple downstream signals, including phosphoinositide 3‐kinase, PKB, p38 MAPK, and ERKs, which then activate endothelial cells. The VEGF/VEGFR‐2 signaling pathway is considered to be the most crucial element in promoting angiogenesis. It is potentially a therapeutic approach for angiogenesis‐related diseases, Alzheimer's disease, Parkinson's disease, and cancer. Hence, numerous VEGFR‐2 inhibitors have been clinically tested and/or validated for the therapeutic use in angiogenesis‐related diseases.^76^, ^152^ Brivanib, a selective dual‐RTK inhibitor, inhibits FGFR and VEGFRs. Li et al.^153^ found that brivanib is an inhibitor of proliferation of microvascular endothelial cells. It could be a new therapeutic option for age‐related macular degeneration.

#### 2.5.6 SFK family

SFK is associated with intracellular signaling cascade,^154^ extracellular stimulation, and activation of various types of receptors including EGFR and VEGF receptors. These receptors translate stimuli into various intracellular signals by triggering SFK. SFK are activated to deliver signals to many pathways. Two of these pathways are associated with major cancer‐related signaling pathways: PI3K, MAPK, and NF‐κB pathway.^155^ The SFKs have been regarded as molecular therapeutic targets. Bosutinib is a competitive inhibitor of Src and ABL TKs and has been shown to bind to the kinase structural domain BCR‐ABL1.^156^ Bosutinib inhibits Src family Src, Lyn, and Hck kinases, as well as c‐Kit and platelet‐derived growth factor (PDGF) receptors.^157^ Now it is used for the treatment of chronic granulocytic leukemia.^158^

#### 2.5.7 BCR‐Abl family

The protein Abl TK reacts with most of the proteins involved in the intracytoplasmic oncogenic pathway when fused to Bcr. Aberrant interactions between Bcr‐Abl oncoproteins favor dimerization or tetramerization, then autophosphorylation. This process causes an increase in the binding sites of phosphorylated tyrosine residues on Bcr‐Abl as well as the SH2 structural domain of other proteins.^159^ Bcr‐Abl has been demonstrated to inhibit apoptosis and promote cell proliferation through the accumulation of genetic abnormalities. Ascitinib is an oral, small‐molecule selective metabolic inhibitor for the treatment of hematologic malignancies.^160^, ^161^ Currently in clinical trial phase I–III. Asciminib acts by selectively binding to the myristoyl pocket of ABL1, but is inactive in vitro against many other kinases as well as G‐protein‐coupled receptors, ion channels, transporters, and nuclear receptors, which may limit its potential for drug targeting.^160^ Primary efficacy results from phase I and III trials demonstrate clinically meaningful efficacy and favorable safety profile of asciminib in patients with chronic myeloid leukemia (CML)‐chronic phase (a phase III, open‐label, randomized study of asciminib, a STAMP inhibitor, vs. bosutinib in CML after two or more prior TKIs).

#### 2.5.8 FLT3 family

FLT3 is a family of RTKs. Its ligand combines with the extracellular domain to activate downstream signaling pathways, such as MAPK and PI3K/PKB signaling pathways that are responsible for hematopoietic cell survival, maturation, and proliferation. The FLT3 receptor is overexpressed in most acute leukemias.^162^ First‐generation FLT3 inhibitors may lead to off‐target toxicity by inhibiting multiple downstream RTKs due to the lack of specificity of FLT3, while second‐generation FLT3 inhibitors can effectively target FLT3 with fewer off‐target effects. A phase III randomized controlled trial found quizartinib to have minimal cutaneous, pulmonary or gastrointestinal side effects and greater myelosuppressive effects than other FLT3 inhibitors.^162^ Gilteritinib usually causes intestinal side effects, such as nausea and diarrhea, and increased bilirubin and transaminases are seen in some patients. Encephalopathy and pancreatitis may occur in severe cases (<1%–2%). Currently, gilteritinib is approved in the United States and Europe for the treatment of adult patients with relapsed or refractory FLT3 mutations in AML.^163^

#### 2.5.9 FGFR family

FGFRs belong to a member of RTK family that exerts a critical role in developing and adult cells.^164^ A variety of cancers, such as uroepithelial, ovarian, and lung adenocarcinoma, are associated with dysregulation of FGFRs. FGFRs are currently regarded as potential pharmaceutical targets for the treatment of various cancers.^165^ A variety of small‐molecule inhibitors have been developed against this family of kinases, and several of which are in clinical trials. Dysregulation of the FGFR TK family is associated with various types of cancers. Derazantinib is an ATP competitive multi‐kinase inhibitor with activity in the low nanomolar range against FGFR1, FGFR2, and FGFR3 kinases.^166^ FGFR2 is the most sensitive kinase, followed by FGFR1. Drazotinib was found to significantly inhibit VEGFR2 phosphorylation through studies in human endothelial cells. Drazotinib significantly reduced tissue permeability and tumor functional vascular system according to animal studies.^166^

#### 2.5.10 BTK family

BTK is a family of the transient erythroblastopenia of childhood TKs. As a non‐receptor intracellular kinase, BTK has recently been found to be in certain tumor isoforms (e.g., multiple myeloma) and other regulating the interaction between malignant clones and the bone marrow microenvironment.^167^ BTK is a key kinase involved in BCR signaling, FcR signaling, Toll‐like receptors (TLRs) signaling, and chemokine receptor signaling. By activating nuclear factor of activated T cells, NF‐κB, and MAPK pathways, it leads to cell proliferation and differentiation, antibody and cytokine production, and expression of co‐stimulatory molecules.^168^, ^169^ Tirabrutinib is a covalent BTK inhibitor used to treat B‐cell lymphoma and chronic lymphocytic leukemia.^170^ Tirabrutinib was approved in Japan in March 2020 for the treatment of relapsed or refractory primary CNS lymphoma. A phase II clinical trial is ongoing to measure the safety and efficacy of tirabrutinib for the treatment of patients with refractory aspergillus.^171^

### 2.6 Tyrosine kinase‐like group

**FIGURE 7:**
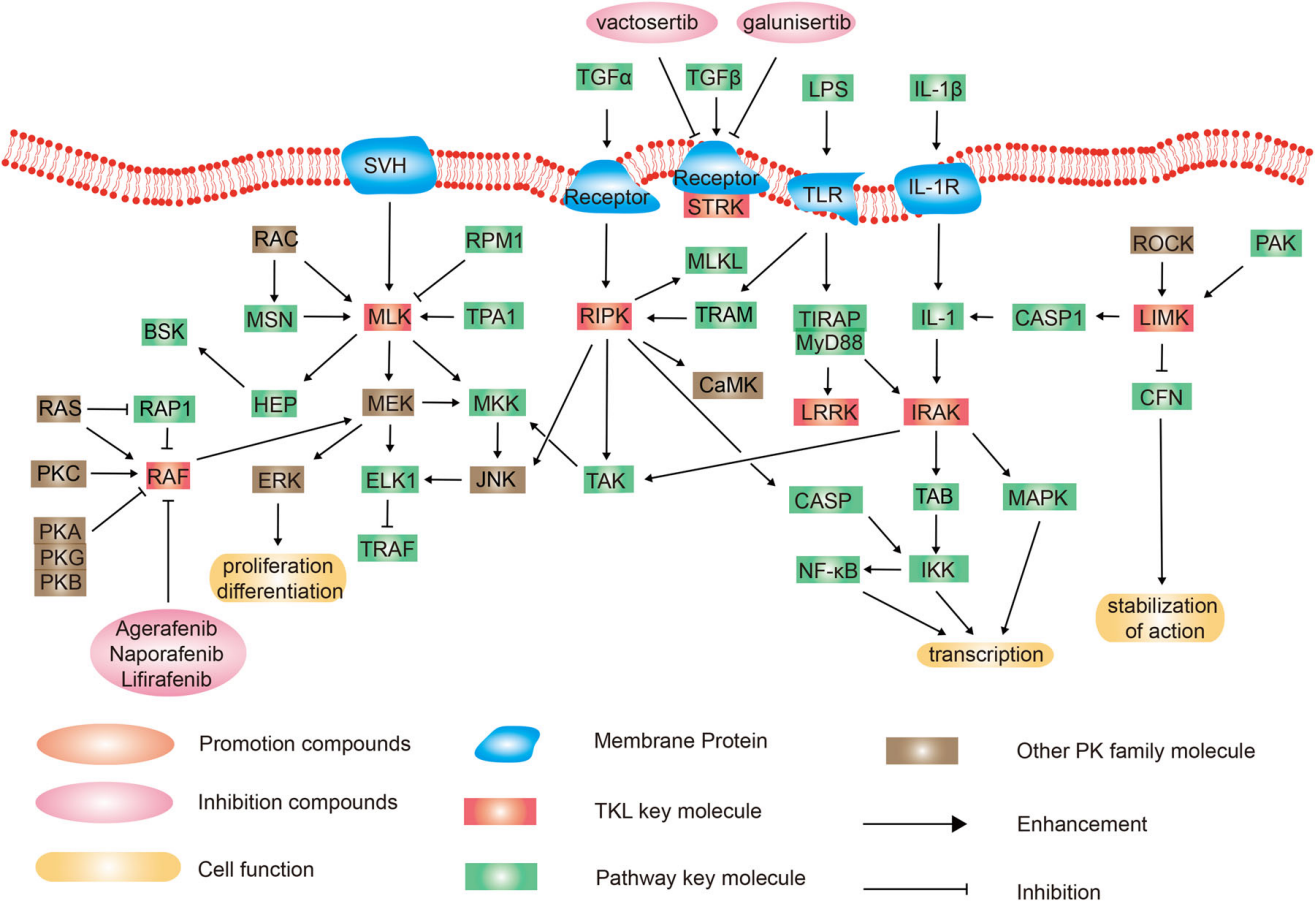
Schematic diagram of protein phosphorylation signaling pathway of tyrosine kinase‐like (TKL) group.

TKL gene family is broadly known to exist in most eukaryotes and is engaged in many biological processes. It contains seven main families including the mixed‐lineage kinase (MLK), LIMK/TESK (LISK), IL‐1 receptor‐associated kinase (IRAK), Raf, receptor‐interacting protein kinase (RIPK), activin and TGF‐receptors (STR), and leucine‐rich repeat kinases (LRRK) (Figure 7).

#### 2.6.1 MLK family

MLKs belong to a member of MAP3K.^172^ Common to all MLK members are the catalytic structural domains containing tagging sequences for serine/threonine (Ser/Thr) and tyrosine (Tyr) kinases, hence the name MLKs. Although MLKs have been demonstrated to be functional Ser/Thr kinases, none of the MLK members has been reported to be Tyr kinase. A possible partial or full member of MLK is functional Tyr kinases for very specific substrates that have not been identified. Another one is that the Tyr kinase activity of MLKs is mediated by specific stimuli; therefore, it is important to recognize these specific stimuli to determine their Tyr kinase activity.^173^ MLK inhibitors have been proposed for a variety of diseases, including metastasis of cancer cells.^174^ Although MLKs are known to be involved in tumorigenic pathways, it is anticipated that clinical application of MLK inhibitors may be challenging because of the existence of multiple downstream targets of MLKs.

#### 2.6.2 LISK family

LIMK is a Ser/Thr kinase and LIMK1 and LIMK2 are the two major subtypes of LIMK.^175^ In the presence of threonine 508 and threonine 505, LIMK1 and LIMK2 are phosphorylated by the upstream kinase ROCK1, tonic dystrophy kinase‐associated Cdc 42‐binding kinase, and p21‐activated kinase 1, 4, or 6.^176^ Activation of kinase is controlled by phosphatases such as p21‐activated kinase 4 (PAK4). Activated LIMK1/2 persistently phosphorylates downstream target protein cofilin at serine 3, resulting in regulated remodeling of actin and microtubulin.^177^ Regulation of LIMK2 localization and stability requires phosphorylation of aurora A and PKC at serine 283, threonine 494, and threonine 505. Thiazolylamide derivatives BMS‐3 and ‐5 can inhibit LIMK1 and LIMK 2 activity.

#### 2.6.3 IRAK family

IRAK1, IRAK2, IRAK‐m, and IRAK4 constitute the IRAK family, which is involved in the activation of TLRs and IL‐1 receptors.^178^ Members of IRAK play either a positive or a negative role in the regulation of inflammation, innate, and adaptive immunity. Accumulating evidence demonstrates that four members of the IRAK family receive activation signals from the junctional molecule MyD88, TIF, and could be auto‐phosphorylated. IRAK plays an essential part in the pathogenesis of inflammatory autoimmune diseases. Inhibition of IRAK has prospective therapeutic benefits. IRAK drugs/inhibitors have a long way to go because of the potential benefits of emerging drugs/inhibitors that target individual IRAKs.^179^

#### 2.6.4 Raf family

The function of RAS proteins is regulated by their post‐translational modifications, their membrane localization, as well as interactions with regulators and effectors. RAS proteins can be modified subtype specifically by distinct mechanisms of isoform specifics to facilitate translocation and plasma membrane localization.^180^ Naporafenib is an ATP‐competitive inhibitor of B‐Raf and C‐Raf. It inhibits not only MAPK signaling activity but also mutated N‐ and Lysyl‐TRNA Synthetase (KRAS)‐driven signaling, and has shown efficacy in many MAPK‐driven human cancer cell lines and xenograft tumors with KRAS, Neuroblastoma RAS [Ras is a protein coding gene's name], and BRAF oncogenes.^181^ Agerafenib eliminates activation of the ERK/MAPK pathway in neuroblastoma. Agerafenib was found to have good cytotoxicity in neuroblastoma mouse models, effectively inhibiting tumor growth and prolonging survival.^182^

#### 2.6.5 RIPK family

RIP kinases RIPK1 and RIPK3 are key cell fate enzymes that play multiple roles in cell death and inflammatory signaling pathways.^183^ RIPK1 mediates NF‐κB activation, thereby enhancing the expression of several pro‐inflammatory cytokines.^184^ During necroptosis, RIPK3 promotes stress‐mediated mitochondrial reactive oxygen species (ROS) generation. Necrosis of cervical cancer cells through phosphorylation of RIPK1, RIPK3, and MLKL, thereby triggering an antitumor immune response in cervical cancer. At present, no ripk1/ripk3/mlkl drugs are currently available for clinical treatment.^185^

#### 2.6.6 STRK family

The TGF‐β superfamily of cytokines plays key roles in the regulation of immune responses that can prevent or promote diseases such as allergies, autoimmunity and cancer. TGF‐β signaling pathway has a major effect in tumor progression, invasion, metastasis, and tumor immunity. Therefore, inhibiting TGF‐β signaling pathway is considered as a prospective anti‐cancer therapeutic strategy.^186^ Vactosertib is a novel inhibitor of TGF‐β type I receptor. Vactosertib acts on the adenosine‐5‐triphosphate‐binding site of the TGF‐β type‐1 receptor thereby inhibiting the phosphorylation of Smad2 and Smad3 substrate proteins.^187^ Currently, small‐molecule inhibitors like vactosertib and galunisertib are being clinically evaluated for the treatment of various cancers such as NSCLC, intestinal cancer, and pancreatic cancer.

#### 2.6.7 LRRK family

LRRK is a serine/threonine kinase belonging to the ROCO protein superfamily. It possesses two homologs leucine‐rich repeat protein kinase, LRRK1 and LRRK2. On the conserved Thr/Ser residues, LRRK2 phosphorylates a subgroup of Rab proteins.^188^, ^189^ Pathogenic mutations in Parkinson's disease enhance LRRK2 protein kinase activity, which stimulates Rab protein phosphorylation.^190^ Novel LRRK2 inhibitors of the aminoquinazoline structure as potential drugs for the therapy of Parkinson's disease.^191^

### 2.7 Atypical group

#### 2.7.1 ATP‐binding cassette transporter 1

ATP‐binding cassette transporter 1 (ABC1) protein kinase family belongs to the atypical protein kinase, which developed before the diversion of archaea and bacteria. ABC1 gene mutations are linked to respiratory defects in microbes and humans.^192^ Complex Q, a complex of enzymes at the mitochondrial inner membrane stabilized by ABC1, is used in yeast and humans for ubiquinone biosynthesis.^193^ Further study is needed to determine how ABC1 functions in the human body. Due to the paucity of research related to ABC1 protein kinase in human diseases, there are no relevant drugs on the market and no ongoing clinical trials.

#### 2.7.2 Alpha kinase

Alpha kinases are an atypical class of protein kinase with a sequence that differs from that of conventional protein kinase families.^194^ A wide variety of cellular processes are mediated by alpha kinases, including translation, Mg(^2+^) homeostasis, intracellular transport, migration, adhesion, and proliferation.^195^ The eEF‐2K kinase is an atypical alpha kinase, which functions as a critical regulator of several cellular processes, and can lead to tumorigenesis by phosphorylating a component of eEF2.^196^, ^197^ The eEF2K is considered a therapeutic target in solid tumors due to its importance in tumorigenesis and tumor progression.^198^ Since alpha kinase is an atypical alpha kinase, “classical” PKIs do not inhibit it, making small‐molecule inhibitor development difficult. There is no alpha kinase inhibitor that was approved by FDA, and no clinical trial was conducted based on the PKIDB.

#### 2.7.3 Pyruvate dehydrogenase kinase

Pyruvate dehydrogenase kinase (PDHK) contributes to aerobic glycolysis by phosphorylating pyruvate dehydrogenase.^199^ PDHK1, PDHK2, PDHK3, and PDHK4 are four distinct PDHK isoenzymes with a high sequence similarity. Pyruvate dehydrogenases (PDKs) are overexpressed in most cancer cells, causing abnormal glucose metabolism. So far, there are three kinds of PDHK inhibitors with different inhibition mechanisms, including PDH2 inhibitor (AED7545 and Nov3r), dichloroacetate (DCA), and N‐terminal ATP‐binding site inhibitor (radicicol). AED7545 is a novel PDHK small‐molecule inhibitor. A study on rats shows that PDHK inhibitor may be a drug to improve blood glucose control in type 2 diabetes.^200^ Nov3r, another PDHK2 inhibitor, can regulate PDH activity and has been proved to have potential therapeutic value in diseases associated with ischemic retinal damage.^201^ DCA, a potential next‐generation oral hypoglycemic drug, by stimulating PDH and increasing peripheral alanine and lactate oxidation, exerts a hypoglycemic effect.^202^ Radicicol is an N‐terminal ATP‐binding site inhibitor, which is considered to have potential therapeutic value in cancer.^203^ Although the number of reported PDHK inhibitors has increased over the years, there is no relevant drug on the market for this target.

#### 2.7.4 Phosphatidylinositol‐3 kinase‐related kinases

Phosphatidylinositol‐3 kinase‐related kinases (PIKKs) are atypical serine/threonine kinases that are related to PI3K. These enzymes participate in a wide range of biological processes, including meiosis, recombination of V(D)J chromosomes, DNA damage detection and repair, and cell cycle progression or arrest. The PIKK family consists of ATR, ATM, DNA‐PKcs, mTOR, and hSMG, that are vital in cancer progression, autophagy, and survival following radiotherapy and chemotherapy.^204^, ^205^, ^206^ Thus, targeting these PIKK kinases in cancer along with chemotherapy and radiation would be helpful for patients. Researchers have developed various PIKK inhibitors^207^ and successfully made into preclinical studies as mono‐ or combinatorial therapies for various human cancers, including alpelisib, apitolisib, buparlisib, and copanlisib.^208^, ^209^, ^210^, ^211^

#### 2.7.5 Right open reading frame kinases

Right open reading frame (RIO) kinases have an atypical fold like protein kinases, which occurs in an ancient group of proteins. There are at least four subfamilies in the RIO kinase family, namely Rio1, Rio2, Rio3, and RioB. RIO1 is an essential member of the RIO family of genes in Saccharomyces *cerevisiae*, which is critical to the proper progression of cell cycle and maintenance of chromosomes.^212^ The FDA approved no RIO inhibitor, and no clinical trial was conducted in the PKIDB. The possible reason for this is that RIO kinase has potential as a therapeutic target, however, its biological function is unknown.

#### 2.7.6 Transcription intermediary factor 1 kinases

In this family, three members, transcription intermediary factor 1 (TIF1)a, h, and g, are involved in transcriptional regulation. Since TIF1a has kinase activity, it is expected that the other TIF1 proteins also possess it due to their high level of conservation. It has been shown that TFIIEa, TAFII28, and TAFII55 can be phosphorylated in vivo. RING finger‐B boxes‐coiled coil (RBCC) and plant homeodomain finger and bromodomain are on one end of the protein, and a bromodomain on the other. It is unknown where the kinase motif is located.^213^ In the PKIDB, there is no TIF1 inhibitor in clinical trial or FDA approval.

### 2.8 Other groups

#### 2.8.1 CK1 family

**FIGURE 8:**
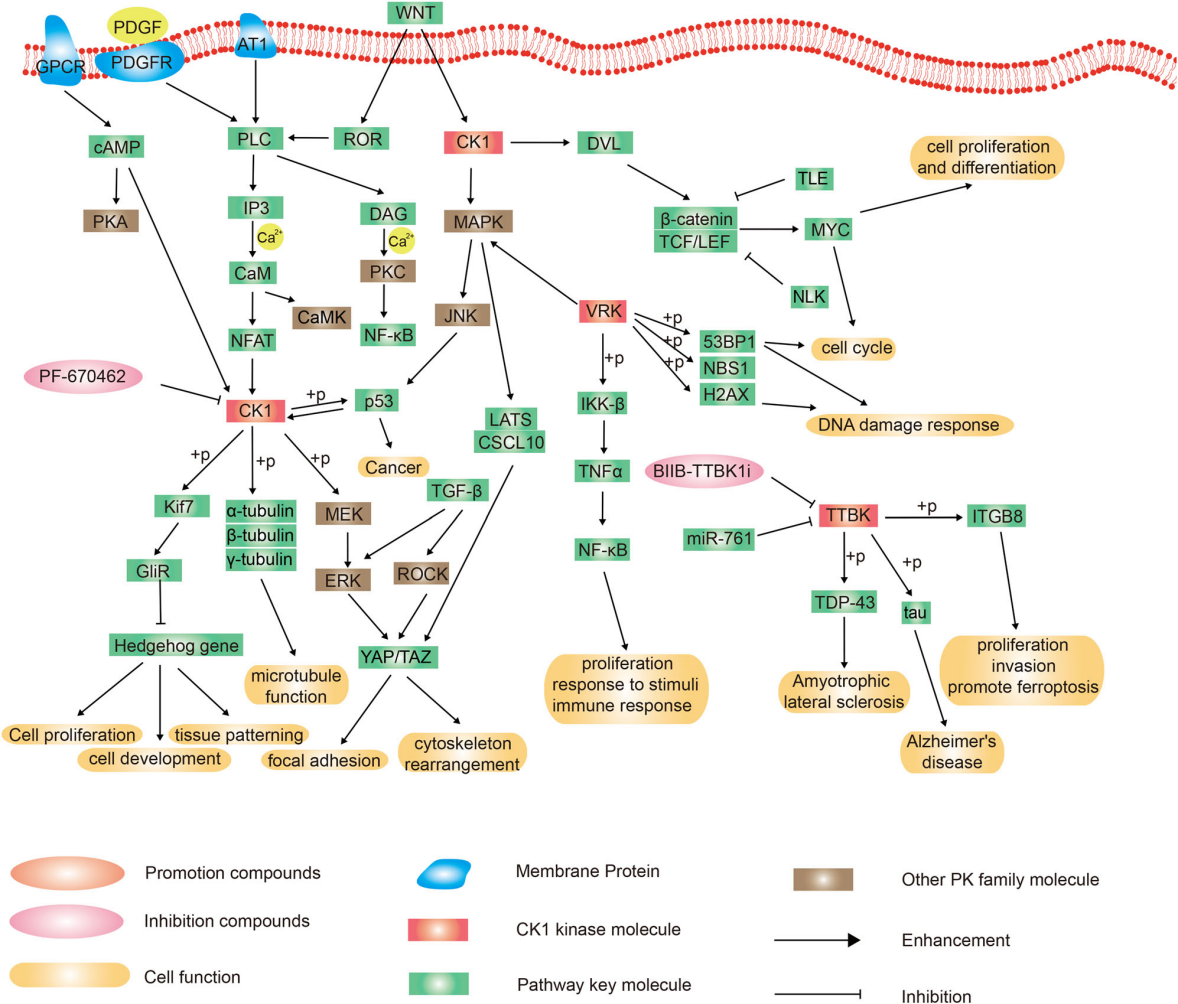
Schematic diagram of protein phosphorylation signaling pathway of other groups.

CK1 kinase regulates mitotic checkpoint signaling transduction, and is involved in DNA repair, apoptosis, p53 pathway, protein translation, circadian rhythm, endocytosis, autophagy, immune response, inflammation, and other related processes. CK1 participates in the signaling transduction process of wingless/integrated (WNT), Hedgehog, NF‐κB, and Yap/Taz (Figure 8).^214^ CK1 activates the WNT signaling pathway and promotes cell cycle and cell proliferation. CK1 is involved in the MEK/ERK/YAP/TAZ signaling pathway to affect focal adhesion formation and actin cytoskeleton rearrangement.^215^ CK1 kinase primarily mediates the phosphorylation of microtubule constituent subunits, such as alpha, beta, and gamma tubulin, leading to the regulation of microtubule polymerization, stability, and spindle dynamics.^216^ p53 is phosphorylated by CK1 and enhances the level of CK1.^217^ Some CK1 inhibitors, such as PF‐670462, is being investigated in clinical trials for the treatment of different neurodegenerative diseases, such as Alzheimer's disease, Parkinson's disease, and amyotrophic lateral sclerosis (ALS).^218^, ^219^, ^220^ However, there is no CK1 inhibitor approved by FDA, in PKIDB or in a clinical trial.

#### 2.8.2 CK1‐TTBK family

TTBK1 phosphorylates TDP‐43, a pathological hallmark of ALS, in cells and animal models.^221^ TTBK is a kinase of tau tubulin, which is a pathological feature of many neurodegenerative diseases, and the level of tau protein correlates with the degree of cognitive impairment.^222^ Besides, TTBK upregulates the level of integrin subunit beta 8 (ITGB8) and affects the proliferation, invasion, and promotes ferroptosis in glioma cells.^223^ Although some TTBK inhibitors have been developed for investigation, there is no TTBK inhibitor approved by the FDA, in the PKIDB, or in a clinical trial.

#### 2.8.3 CK1‐VRK family

In cancer cells, VRK activates TNFα/NF‐κB signaling by phosphorylating IKKβ, and regulates cell cycle and DNA damage response (Figure 8).^224^, ^225^ It was demonstrated that VRK2 is a specific therapeutic target, and its inhibitor combined with PD‐1 blockade may be effective in improving immunotherapy in patients with cancers.^226^

#### 2.8.4 DNA‐dependent protein kinase

DNA‐dependent protein kinase (DNA‐PK) is a type of kinase that phosphorylates nuclear DNA helicase II/RNA helicase A and hnRNP proteins in an RNA‐dependent manner.^227^ The catalytic subunit of DNA‐PKs regulates the classical non‐homologous end joining (c‐NHEJ) pathway, which is an important repair mechanism for DNA double‐strand breaks (DSBs) related to DNA damage and cell survival.^228^ In recent years, some DNA‐PK drugs have been researched and developed for the treatment of tumors, such as AZD‐7648,^229^ peposertib, and others. Peposertib was reported as an oral DNA‐PK in 2020 and achieved satisfactory tumor‐inhibiting effects in animal experiments.^230^ As a typical representative of DNA‐PK, peposertib can inhibit DNA double‐strand repair by inhibiting DSBs, enhancing the killing effect of DNA DSBs induced by radiotherapy and chemotherapy.^231^

#### 2.8.5 Immune checkpoint inhibitors

The use of immune checkpoint inhibitors (ICIs) has ushered an immune era of tumor treatment; however, the effective response rate is unsatisfactory for the cancers with low immunogenicity. The inhibition of some pathways can enhance the immune response of cells, such as ADP‐ribose polymerase inhibitor (PARPi), which can prevent single‐strand break DNA repair, activate antigen‐presenting cells, and upregulate tumor programmed death ligand‐1 (PD‐L1).^232^

Prexasertib, rabusertib, and adavosertib are the latest ICIs that are in clinical trials.^233^ Prexasertib has a good application value in ovarian neoplasms because there is evidence of cytotoxic T‐cell activation in the blood of patients when it is combined with PD‐L1 inhibitors.^234^ Rabusertib showed a good synergistic activity in platinum‐resistant breast cancer cells.^235^

#### 2.8.6 Aurora kinase B

Aurora kinase B (AURKB) inhibitor is a kind of cell cycle inhibitors^236^ and there are two AURKB inhibitors, named barasertib and danusertib, in PKIDB.^5^ It is reported that barasertib, which is discovered in 2020,^237^ can attenuate fibroblast activation and pulmonary fibrosis induced by TGF‐α.^238^ Besides, danusertib is effective in the treatment of several tumors such as adenocarcinoma and hepatocellular carcinoma due to its effect on cell cycle arrest.^239^ In addition to these two drugs, there is another one, ilorasertib, which has the inhibitory activity on AURKB and VEGF.^240^

#### 2.8.7 Multi‐kinase inhibitor

There are also many multi‐kinase inhibitors that achieve different effects depending on the kinase they inhibit. For example, ilorasertib can inhibit AURKB and VEGF; rigosertib can simultaneously inhibit CDK1, RAS, and MEK1 to affect cell cycle, cell proliferation, and survival.^241^, ^242^, ^243^ Inhibitors with multiple targets can achieve better blocking effects, resulting in more effective therapeutic effects; but at the same time, there will also be a problem of increased side effects due to simultaneous blocking of multiple targets.^244^

## 3 DISCUSSION

At present, including the earliest FDA approved imatinib, the vast majority of PKIs mainly focus on TK and TKL groups. Of the 303 drugs we discussed, more than half (63.4%) were concentrated in these two groups, and 18.2% of the drugs belonged to the AGC‐RGC group, 11.2% of the drugs belonged to the CMGC group, 2.3% of the drugs belonged to the STE‐MAPKs group, and 1.0% of the drugs belonged to the atypical group. The AGC group is represented by the cAMP‐dependent protein kinase PKA, the cGMP‐dependent protease PKG, and the calcium‐ and phospholipid‐dependent protein kinase PKC, and is characterized by activation by second messengers such as cAMP, cGMP, diacylglycerol, and Ca^2+^.^10^ TKs can transfer γ‐phosphate on ATP to protein tyrosine residues, catalyze the phosphorylation of protein tyrosine residues of various substrates, and play an important role in cell growth, proliferation, and differentiation.^245^ TKIs, on the other hand, further inhibit the growth and proliferation of tumor cells or other proliferative diseases, and promote apoptosis mainly by inhibiting cell signal transduction.^245^, ^246^ One of the advantages of TKI for antitumor is its anti‐angiogenesis effect. Because tumors are mostly rich in blood supply, TKI's inhibitory effect on neo‐angiogenesis can reduce unnecessary side effects.^247^ Side effects are a constant problem in drug development. The known adverse events during TKI development include leukopenia, thrombocytopenia, anemia, neutropenia, hypertension, abnormal liver function, oral mucosal inflammation, fatigue, sensory nerve abnormalities, thyroid dysfunction, hypopigmentation, proteinuria, and gastrointestinal symptoms.^248^ Many new TKIs have been improved in terms of enhancing targeted delivery, prolonging circulation time, improving targeted delivery efficiency, and reducing normal tissue damage to improve their effects.^249^ The main focus of CMGC histone kinase drugs is the CDK, MAPK, and GSK3 families.^250^ CDK mainly affects the cell cycle, MAPK is related to cell division, and GSK3 family is related to glycogen synthesis.^8^ These pathways have effects on cell proliferation and active division, and are one of the main development directions of new PKI drugs.^251^ Table 1 briefly summarized the protein kinase group, family, target, disease, and years approved by FDA in recent 5 years.

**TABLE 1:** Proteins kinase inhibitor (PKI) drugs approved by Food and Drug Administration (FDA) in recent 5 years

Approved years	Protein kinase	Group	Family	Targets	Disease
2017	Abemaciclib	CMGC	CDKs	CDK4/6	Breast neoplasms, osteosarcoma
	Neratinib	CMGC	CDKs	ErbB2/HER2	Breast cancer
	Ribociclib	CMGC	CDKs	CDK4/6	Breast cancer
	Brigatinib	TK	Raf	ALK	Non‐small‐cell lung cancer
	Midostaurin	TK	FLT3	Flt3	Acute myeloid leukemia
	Acalabrutinib	TKs	BTK	BTK	Chronic lymphocytic leukemia, mantle cell lymphoma
2018	Netarsudil	AGC	ROCK	ROCK1/2	Treat glaucoma
	Binimetinib	STE‐MAPKs	Ste7/MAP2K	MEK1/2	Metastatic colorectal cancer, advanced melanoma
	Dacomitinib	TK	EGFR	EGFR	Non‐small‐cell lung cancer
	Fostamatinib	TK	SYK	Syk	Immune thrombocytopenia
	Gilteritinib	TK	FLT3	Flt3	Acute myeloid leukemia
	Larotrectinib	TK	TRK	TRKA/B/C	Papillary thyroid cancer
	Lorlatinib	TK	ALK	ALK	Non‐small‐cell lung cancer
	R406	TK	SYK	Syk	Leukemia, inflammation, glomerulonephritis
	Encorafenib	TKL	Raf	B‐Raf	Colorectal cancer, melanoma
	Baricitinib	TKs	JAK	JAK1/2	Rheumatoid arthritis, moderate to severe atopic dermatitis
2019	Erdafitinib	TK	FGFR	FGFR1/2/3/4	Urothelial cancer
	Fedratinib	TK	JAK	JAK2	Myelofibrosis, myeloproliferative neoplasm
	Pexidartinib	TK	CSF	CSF1R	Tenosynovial giant cell tumors
	Upadacitinib	TK	JAK	JAK1	Psoriatic arthritis, rheumatoid arthritis
	Zanubrutinib	TK	BTK	BTK	Waldenström macroglobulinemia, mantle cell lymphoma
	Entrectinib	TKL	Raf	TRKA/B/C, ROS1	Non‐small‐cell lung cancer
2020	Selumetinib	STE	Ste7/MAP2K	MEK1/2	Neurofibromatosis type 1
	Capmatinib	TK	RET	c‐MET	Non‐small‐cell lung cancer, glioblastoma, liver cancer, malignant melanoma, breast cancer, colorectal cancer, head and neck cancer
	Pemigatinib	TK	FGFR	FGFR2	Cholangiocarcinoma, myeloid/lymphoid neoplasms
	Pralsetinib	TK	RET	RET	Non‐small‐cell lung cancer, papillary thyroid cancer, and medullary thyroid carcinoma
	Ripretinib	TK	SFK	Kit, PDGFRα	Gastrointestinal stromal tumour
	Selpercatinib	TK	RET	RET	Non‐small‐cell lung cancer, thyroid cancer, and medullary thyroid cancer
	Tucatinib	TK	EGFR	ErbB2/HER2	Breast cancer and colorectal cancer
	Avapritinib	TKs	SFK	PDGFRα	Systemic macrocytosis
2021	Belumosudil	AGC	ROCK	ROCK1/2	Chronic graft‐versus‐host disease
	Umbralisib	AGC	PI3K	PI3K	Non‐Hodgkin lymphoma
	Trilaciclib	CMGC	CDK	CDK4/6	Small‐cell lung cancer
	Asciminib	TK	BCR‐ABL	ABL	Chronic myeloid leukemia
	Infigratinib	TK	FGFR	FGFR	Cholangiocarcinoma
	Mobocertinib	TK	EGFR	EGFR	Non‐small‐cell lung cancer
	Tepmetko	TK	RET	MET	Non‐small‐cell lung cancer
	Sotorasib	TKL	RAF	KRAS	Non‐small‐cell lung cancer
2022	Dabrafenib	TKL	RAF	BRAF	Mutated melanoma, non‐small‐cell lung cancer, mutated anaplastic thyroid cancer

Abbreviations: AGC, protein kinase A, G, and C; ALK, anaplastic lymphoma kinase; BTK, Bruton's tyrosine kinase; BCR‐Abl, breakpoint cluster region‐Abelson fusion protein; CDK, cyclin‐dependent kinases; CMGC, **c**yclin‐dependent kinases, **m**itogen‐activated protein kinases, **g**lycogen synthase kinases, and **C**dc2‐like kinases; CSF, colony‐stimulating factor; EGFR, epidermal growth factor receptor; FGFR, fibroblast growth factor receptor; FLT, FMS‐like tyrosine kinase; JAK, Janus kinase; MAPK, mitogen‐activated protein kinase; PDGF, platelet‐derived growth factor; ROCK, rho‐associated coiled‐coil‐containing kinase; SFK, Src‐family protein tyrosine kinase; SYK, spleen tyrosine kinase; TK, tyrosine kinase; TKL, tyrosine kinase‐like.

*Source*: FDA website https://www.fda.gov/

There are many studies, reports, and reviews that focus on protein phosphorylation, some provide updated PKI classification, and some provide good research depth and content. This review starts with the latest drugs under clinical trials or under clinical use, and focuses on their targets, which can reflect the frontier trends of intervention therapy targeting protein phosphorylation. In addition, the preliminary reasons for the groups and families with no or few new drugs are also analyzed, and suggestions and directions are provided for the development of drugs in this area. Because protein phosphorylation kinases are very complex, involving a lot of pathways, drugs, and diseases, this review cannot cover all the content, and there is no major improvement and innovation on the previous classification methods of protein kinases. In the following review, we will consider an improvement to the existing classification of protein phosphorylation kinases, and provide a novel framework for classification of protein kinases.

## 4 CONCLUSION

There are many kinds of human protein kinases that functionally promote or inhibit each other, constituting an extremely complex signaling transduction network and information regulation network in human beings. For these kinases, researchers have developed a variety of specific or non‐specific inhibitory drugs to achieve the purpose of disease treatment. The current status of PKIs development is that PKIs are mainly focused on antitumor treatment, and a few drugs are used for the treatment of Alzheimer's disease, immune diseases, proliferative diseases, and even COVID‐19. Now there are still some problems in the development of new PKIs, such as high electrophilic reactivity, non‐specific cytotoxicity, and target cysteine mutation disadvantages, which lead to some less therapeutic effects and unsatisfactory side effects. With the continuous efforts of scientists in recent years, new drugs with fewer side effects have been developed. We expect to see a bright future of drugs targeting protein phosphorylation to benefit patients.

## AUTHOR CONTRIBUTIONS

*Conceptualization*: K.P., Z.‐S.C., and C.‐H.H. *Literature review*: K.P., W.W., J.‐X.Q., Z.‐D.S., L.H., Y.‐Y.M., Z.‐X.W., H.X., D.P., Z.‐S.C., and C.‐H.H. *Writing—original draft preparation*: K.P., W.W., and J.‐X.Q. *Writing—review and editing*: Z.‐S.C. and C.‐H.H. *Supervision*: Z.‐S.C. and C.‐H.H. All authors read and approved the final manuscript.

## CONFLICT OF INTEREST

Author Zhe‐Sheng Chen is an editorial board member of MedComm. Author Zhe‐Sheng Chen was not involved in the journal's review of, or decisions related to, this manuscript. The other authors declared no conflicts of interest.

## ETHICS STATEMENT

Not applicable.

## Supporting information

Table S1: Proteins kinase inhibitor (PKI) drugs under clinical trials in Protein Kinase Inhibitor Database (PKIDB). *Source*: PKIDB website https://www.icoa.fr/pkidb/

## Data Availability

Not applicable.
